# Influence of Fermentation Water on Stable Isotopic D/H Ratios of Alcohol Obtained from Concentrated Grape Must

**DOI:** 10.3390/molecules25143139

**Published:** 2020-07-09

**Authors:** Matteo Perini, Andrea Failoni, Marco Simoni, Agostino Tonon, Federica Camin

**Affiliations:** 1 Fondazione Edmund Mach (FEM), Via Mach 1, 38010 San Michele all’Adige (TN), Italy; matteo.perini@fmach.it (M.P.); marco.simoni@fmach.it (M.S.); agostino.tonon@fmach.it (A.T.); 2 Center Agriculture Food Environment (C3A), University of Trento, via Mach 1, 38010 San Michele all’Adige (TN), Italy; andrea.failo7@gmail.com

**Keywords:** concentrated grape must, SNIF-NMR analysis, dilution, normalisation, GMWL

## Abstract

According to Organisation Internationale de la vigne et du vin (OIV) standards, when analysing the stable isotope ratio of deuterium to hydrogen D/H at the methyl (I) and methylene (II) site of ethanol from concentrated must, a dilution with tap water is needed in order to carry out the alcoholic fermentation. This dilution causes a partial transfer of water hydrogens to the sugar, and this affects the (D/H)_I_ and (D/H)_II_ isotopic values of ethanol, which need to be normalised through specific equations based on the analysis of water δ^18^O or δ^2^H. The aim of this study was to evaluate the effectiveness and correctness of these equations experimentally. Grape, cane, and beet sugar, as well as grape must were diluted with water with increasing H and O stable isotope ratios, fermented, and analysed. SNIF-NMR and IRMS techniques were applied following the respective OIV methods. The equations based on the δ^2^H analysis of the diluted sugar/must solutions proved to be reliable in all the cases, although it is not an OIV standard. When using the equations based on the values of δ^18^O of the diluted solution, data normalisation was reliable only in cases where the water used for dilution had not undergone isotopic fractionation due, for example, to evaporation. In these cases, δ^2^H should be analysed.

## 1. Introduction

Stable isotope ratio analysis (SIRA) is a well known and powerful technique to detect the counterfeiting of different food matrices, from wine and grape must to olive oil [1]. The European Committee for Standardization (CEN), the Organisation Internationale de la vigne et du vin (OIV), and the Association of Official Agricultural Chemists (AOAC) have recognized this ability and have released several official methods based on this type of analysis [2]. 

In the case of grape derivatives (wine, must, and vinegar) they are based on the site-specific analysis of the isotopic ratios of deuterium and hydrogen of the methyl (D/H)_I_ and methylene (D/H)_II_ sites of ethanol (method OIV-MA-AS311-05) or of the methyl site of acetic acid (D/H)_CH3_ (EN 16466-1) using ^2^H-SNIF-NMR (Site Specific Natural Isotope Fractionation-Nuclear Magnetic Resonance) and ^13^C/^12^C ratio analysis (expressed as δ^13^C ‰) of ethanol (OIV-MA-AS312-06) or of acetic acid (EN 16466-2 and OIV 510/2013). The aim of these specific methods is to identify the addition of exogenous sugar (e.g., beet or cane) or synthetic ethanol or acetic acid to the declared grape products, a practice that is considered illegal outside of specific wine-growing regions (e.g., France, Germany, or the United Kingdom) that may suffer from a lack of sun hours during the grape-ripening period [3].

Another method based on the IRMS (Isotope Ratio Mass Spectrometry) analysis of the ^18^O/^16^O ratio (δ^18^O ‰) in water is useful to identify the dilution of wine (OIV-MA-AS2-12) and vinegar (EN 16466-3 and OIV 511/2013) with water. 

This practice, too, is considered a fraud according to European Regulations 479/2008 and 1234/2007 (European Commission 2007, 2008a); such legislation, following the OIV definitions of the vitivinicultural products, considers wine as a product obtained exclusively from the alcoholic fermentation of fresh grapes, whether crushed or not, or grape must (OIV Code Sheet-Issue 2015/01 I.1.3-1) and wine vinegar as a product obtained exclusively by the acetic fermentation of wine (OIV Code Sheet-Issue 2015/01 I.1.6-11). 

To carry out the site-specific SNIF-NMR isotopic analysis of deuterium in the ethanol of concentrated musts (CM) and rectified concentrated musts (CRM), the official OIV method (MA-AS311-05) provides for an initial dilution of the medium with water before carrying out fermentation. This is due to excessive sugar concentration that could cause osmotic stress in the yeast and, therefore, hinder the fermentation [4]. The fermentation water used for dilution is tap water with ^2^H/^1^H and ^18^O/^16^O different from those of the original must water and also, as demonstrated by Singh et al. [5], differing as to where (geographical origin) and when (season) it was collected. As reported in the WaterIsotope database administered by Gabriel Bowen (data available in the database http://wateriso.utah.edu), tap water can vary from strongly negative to positive values (e.g., the ground water with ^18^O = +6‰ from Egypt [6]). 

During fermentation, the water used for dilution causes a partial transfer of hydrogen to sugar, which will affect the isotopic values D/H of ethanol [7]. It is well known that D/H of ethanol and especially (D/H)_I_ memorizes the D/H of the unexchangeable H of sugar, which are different for grape, cane, and beet sugar and, therefore, allows for the detection of the addition of exogenous sugars to grape products. After dilution with water, due to the partial transfer of H, the final D/H is influenced also by the D/H of water and is, therefore, unreliable. To avoid this, it is necessary to correct the D/H values of ethanol. This correction, named in the OIV method as normalisation, is performed by applying specific equations suggested by Martin et al. [8].

This study aimed at evaluating the effectiveness and correctness of the equations used to normalise the D/H values of alcohol with respect to the contribution of tap water, as there are currently no experimental works in the literature verifying this.

For the purpose of this study, sugars of different origins (beet, cane, and grapes) were considered, which were then fermented in five waters with different δ^18^O (from about −10‰ to about +10‰) and δ^2^H values (from −60‰ to about −5‰); finally, the (D/H)_I_ and (D/H)_II_ ratios of the ethanol produced were measured and corrected by using δ^18^O or δ^2^H. The correctness of the equations was verified by comparing the results for ethanol from the same sugar fermented in water with increasing δ^18^O and δ^2^H values. If the corrected (D/H)_I_ and (D/H)_II_ values did not change with the values of the water, then the equations were correct.

For further confirmation, the isotopic values of (D/H)_I_ and (D/H)_II_ of ethanol of three different grape musts were also analysed after fermentation and distillation. The musts were analysed and subsequently concentrated and diluted with four waters with increasing δ^18^O and δ^2^H values. The isotopic values of (D/H)_I_ and (D/H)_II_ of ethanol from concentrated must, normalised with respect to the δ^18^O or δ^2^H of the diluted must samples, were then compared with those of the original must.

### Rationale

The OIV MA AS-311-05 method prescribes the application of specific equations, explained by Martin et al. [8], to normalise the isotopic values.

In these equations, parameters (D/H)_I_ and (D/H)_II_, measured, respectively, in the methyl and methylene sites of ethanol, are expected to have hydrogen coming from fermentation water for a percentage of 19% and 78%, respectively.

The reference water used for normalisation is the V-SMOW standard, with D/H equal to 155.76 ppm [8]. From these values the following equations are obtained:

(1) $${\left(\frac{D}{H}\right)}_{I}^{Norm.V-SMOW}={\left(\frac{D}{H}\right)}_{I}-0.19\times \left[{\left(\frac{D}{H}\right)}_{W}^{S}-155.76\right]$$

(2) $${\left(\frac{D}{H}\right)}_{II}^{Norm.V-SMOW}={\left(\frac{D}{H}\right)}_{II}-0.78\times \left[{\left(\frac{D}{H}\right)}_{W}^{S}-155.76\right]$$

where:

- 0.19 refers to the percentage of hydrogen in the methyl site of ethanol that derives from the tap water used for fermentation, influencing the ratio (D/H)_I_
- 0.78 refers to the percentage of hydrogen in the methylene site of ethanol that derive from the tap water used for fermentation, influencing the ratio (D/H)_II_

${\left(\frac{D}{H}\right)}_{W}^{S}\;$ = D/H of Sample Water, i.e., of the water of the diluted solutions, refers to the isotopic ratio of deuterium (D/H) in the diluted must water expressed in ppm instead of delta per mill.

The latter can be calculated using the following equation as reported in the OIV method:

(3) $${\left(\frac{D}{H}\right)}_{W}^{S}=155.76\times \left[\frac{\left(8\times \delta^{18}O+10\right)}{1000}+1\right]$$

δ^18^O is measured on the diluted must by determining the ^18^O/^16^O isotopic ratio of the water. When measuring δ^2^H of water, it replaces the equation (8 × δ^18^O + 10). This equation was defined by the geochemist Harmon Craig [9] on the basis of the correlation linking the values of δ^18^O with those of δ^2^H of a large number of precipitation samples:

(4) $$\delta^{2}H=8\times \delta^{18}O+10$$

This correlation defined as the Global Meteoric Water Line has been confirmed by Rozanski et al. [10] using a larger dataset of precipitation water.

## 2. Results and Discussion

### 2.1. (D/H)_I_ and (D/H)_II_ Isotopic Ratios of Different Sugars

Table 1 shows the δ^18^O and δ^2^H values of water of diluted sugar samples and of (D/H)_I_ and (D/H)_II_ of ethanol obtained after fermentation of the three different sugars, both not corrected and after correction (Equations (1) and (2)) using, for the calculation of ${\left(\frac{D}{H}\right)}_{W}^{S}\;,\;$ both δ^18^O (Equation (5)) and δ^2^H (Equation (6)). 

The δ^18^O and δ^2^H values of diluted sugars are almost the same as those of the used dilution water because the sugar did not contain water.

As expected [11], the (D/H)_I_ values confirmed their dependence on the botanical source of sugar (cane vs. beet vs. grape), whereas the (D/H)_II_ values depend on the isotopic composition of the water used in the fermentation medium. 

Beet sugar has the lowest (D/H)_I_ values, both corrected and uncorrected, grape sugar recorded intermediate values, whereas cane sugar showed the highest values, in agreement with what has been reported in the literature [11]. The uncorrected (D/H)_I_ values varied from around 92 to around 108 ppm, depending on the sugar type, and averaged 1.8 ppm on the basis of the values of dilution water used. The values of (D/H)_II_, on the other hand, did not vary a lot with the sugar type (e.g., from 120.3 ppm to 123.8 ppm for uncorrected values with a the same δ^18^O of dilution water) but changed an average of 7.3 ppm on the basis of the value of water used for dilution. Indeed, as explained above, (D/H)_II_ is more influenced by the δ^18^O and δ^2^H of the dilution water than is (D/H)_I_. This is caused by the fact that 78% of the H of the methylene site (D/H)_II_ derives from fermentation water, while only 19% of the H of the methyl site (D/H)_I_ is derived from the fermentation water [8].

The values of (D/H)_I_ and (D/H)_II_ reported in Table 1 were then normalised with respect to the standard water V-SMOW with a certified D/H value equal to 155.76 ppm, using the equation explained in the Rationale section. 

The ${\left(\frac{D}{H}\right)}_{W}^{S}$ value was obtained in the following two ways:

(5) $${\left(\frac{D}{H}\right)}_{W}^{S}=155.76\times \left[\frac{\left(8\times \delta^{18}O+10\right)}{1000}+1\right]$$

(6) $${\left(\frac{D}{H}\right)}_{W}^{S}=155.76\times \left[\frac{\delta^{2}H}{1000}+1\right]$$

where δ^18^O and δ^2^H are the data of the water of the diluted sugar samples.

**Figure 1:**
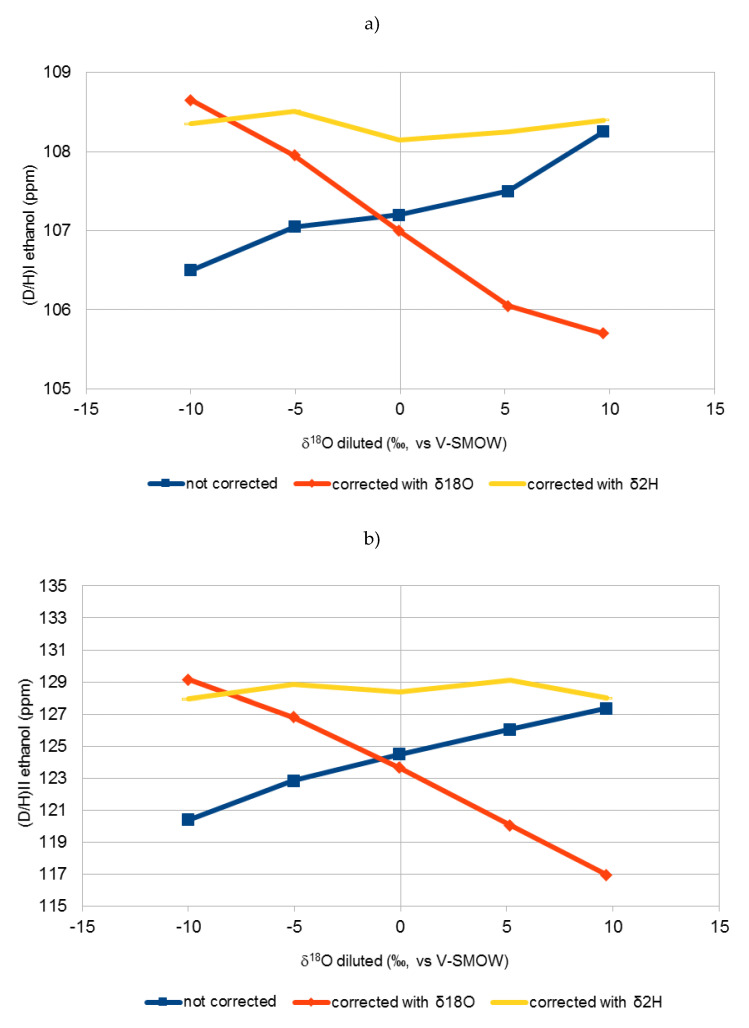
Variation of uncorrected (D/H)_I_ (**a**) and (D/H)_II_ (**b**) values of ethanol vs. δ^18^O of water of diluted cane sugar samples (red line) and effect of the correction on the (D/H)_I_ (**a**) and (D/H)_II_ (**b**) of ethanol (Equations (1) and (2)) using δ^18^O (Equation (5)) (blue line) and δ^2^H (Equation (6)) (yellow line), values reported in Table 1 of the diluted sugar samples. Each point is the mean of two samples.

**Figure 2:**
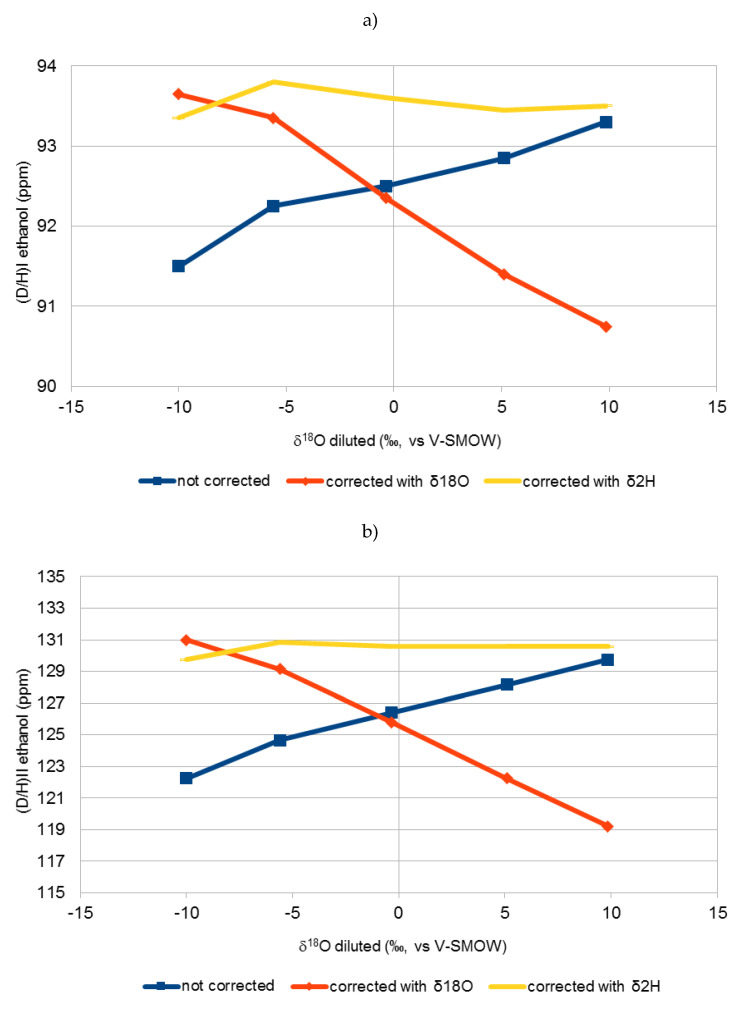
Variation of uncorrected (D/H)_I_ (**a**) and (D/H)_II_ (**b**) values of ethanol vs. δ^18^O of water of diluted beet sugar samples (red line) and effect of the correction on the (D/H)_I_ (**a**) and (D/H)_II_ (**b**) of ethanol (Equations (1) and (2)) using δ^18^O (Equation (5)) (blue line) and δ^2^H (Equation (6)) (yellow line), values reported in Table 1 of the diluted sugar samples. Each point is the mean of two samples.

**Figure 3:**
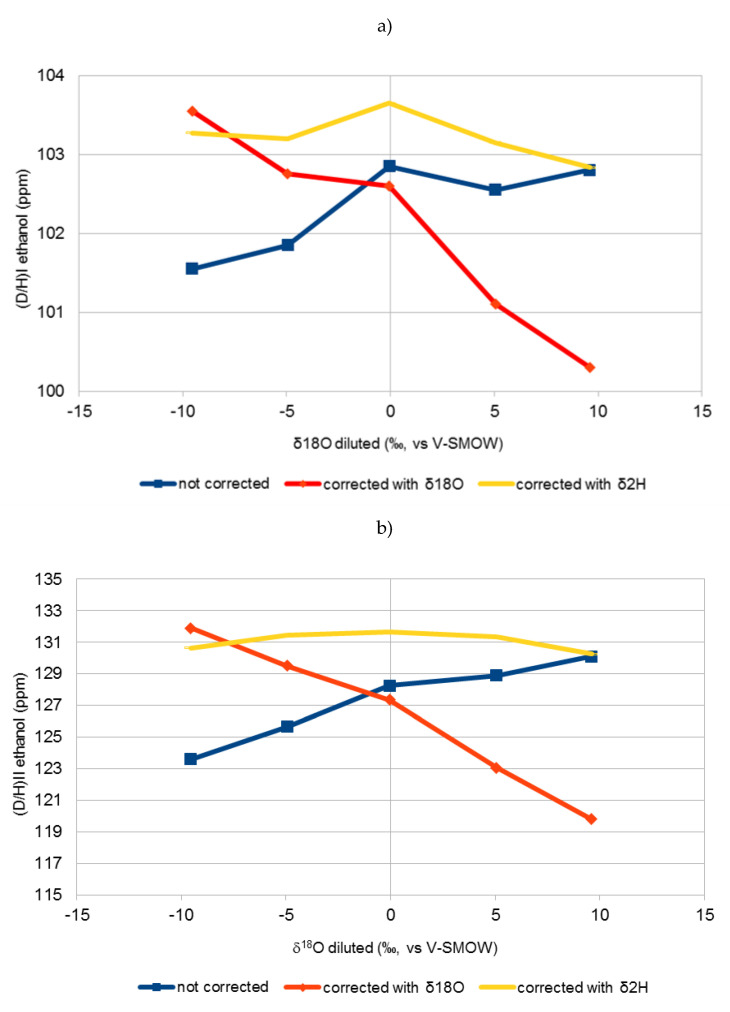
Variation of uncorrected (D/H)_I_ (**a**) and (D/H)_II_ (**b**) values of ethanol vs. δ^18^O of water of diluted grape sugar samples (red line) and effect of the correction on the (D/H)_I_ and (D/H)_II_ of ethanol (Equations (1) and (2)) using δ^18^O (Equation (5)) (blue line) and δ^2^H (Equation (6)) (yellow line), values reported in Table 1 of the diluted sugar samples. Each point is the mean of two samples.

Figure 1, Figure 2 and Figure 3 show the variation of the uncorrected and corrected (D/H)_I_ (**a**) and (D/H)_II_ (**b**) values of ethanol with δ^18^O values of water of diluted sugar samples. Equations (1) and (2) was used for correction purposes taking into account, for calculation of ${\left(\frac{D}{H}\right)}_{W}^{S}$, both δ^18^O (Equation (5)) and δ^2^H (Equation (6)).

It is clear that correction of Equations (1) and (2) using the δ^18^O value of water (Equation (5)) were not effective in normalising all the results. In fact, a very large difference was obtained between the (D/H)_I_ and (D/H)_II_ values of the same matrix (e.g., beet sugar) fermented with waters with different δ^18^O. These differences were even greater in the corrected data than in the non-corrected data. Equations (1) and (2) seem to be effective only if dilution water has a δ^18^O between −10 and −5‰, which is the usual range of values for the tap water most commonly found in European areas.

On the other hand, by applying Equations (1) and (2) using the δ^2^H values of diluted sugars (Equation (6)), there is no significant difference (lower than 1 ppm) between the values of (D/H)_I_ and (D/H)_II_ of the same matrix fermented with different waters, which shows the effectiveness of this equation in all cases.

The (D/H)_I_ and (D/H)_II_ values of ethanol, corrected using Equation (6) in Equations (1) and (2), are not significantly different from those obtained using Equation (5) in Equations (1) and (2) for values of δ^18^O of water of the sugar solution ranging between −10 and −5 ‰ and from those of uncorrected values for δ^18^O of water higher than +5 ‰.

Equations (1) and (2), reported in the official OIV method, are based on the prediction of δ^2^H of water from δ^18^O values deriving from the Global Meteoric Water Line (GMWL). For δ^18^O higher than −5 ‰, coming in this case mainly from evaporated water, this correlation did not follow the GMWL anymore.

**Figure 4:**
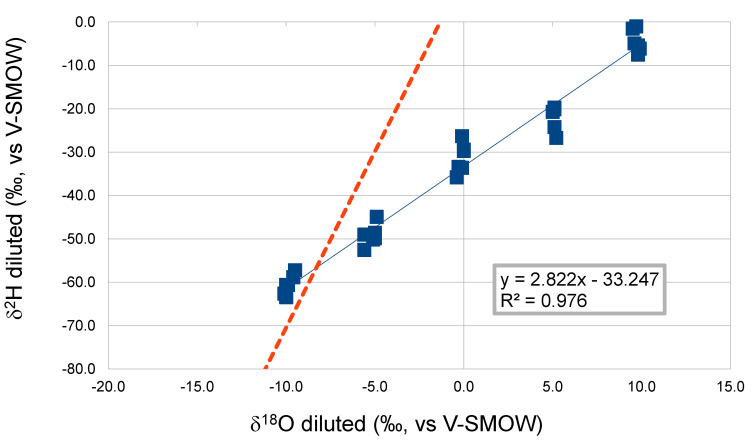
Correlation between δ^18^O and δ^2^H of diluted sugar solutions (data for all three types of sugars). The Global Line of Meteoric Waters is the dotted one.

During evaporation, isotopic fractionation occurs that causes a deviation of the δ^18^O vs. δ^2^H correlation compared to the Global Meteoric Waters Line [9,12], as is also evident in Figure 4. The correlation is still a highly significant linear correlation (R^2^ close to 1) but with different slope and intercept to those of the GMWL.

A similar trend was reported by Raco et al. and Ingraham et al. [13,14] who analysed several must and grape berry waters. A clear deviation of the correlation between δ^18^O and δ^2^H values was reported not only with respect to the global meteoric water line (GMWL) [9] but also according to Raco et al. with respect to the Italian meteoric water line (IMWL) [15]. Additionally, for Ingraham et al. [14], the δ^18^O and δ^2^H correlation line of the grape berries water has a slope of 2.8, indicating that it was controlled by various isotopic kinetic effects on water loss imparted by the different surface transmissivities of the berry, as well as isotopic exchange with ambient water vapor. Thus the slope found here is the same of that observed in vegetable water that underwent evapotranspiration [14].

To conclude, when the water used for dilution suffered isotopic fractionation due, for example, to evaporation and thus the GMWL was no longer valid, i.e., in this study for δ^18^O higher than −5 ‰, Equation (6) should be used for the calculation of ${\left(\frac{D}{H}\right)}_{W}^{S}\;$ in the Equations (1) and (2). Therefore, δ^2^H of diluted must should be analysed, even if this analysis has not yet been recognized as an official standard by OIV.

### 2.2. (D/H)_I_ and (D/H)_II_ Isotopic Ratios of Grape Must

In this case, the reference (D/H)_I_ and (D/H)_II_ values of fresh musts are available: must 1 (99.9 and 122.5 ppm), must 2 (101.0 and 128.4 ppm), and must 3 (101.8 and 127.9 ppm). Table 2 showed how, even in this case, the correction by Equations (1) and (2) using Equation (5) (based on δ^18^O analysis of diluted must) is acceptable for dilutions with tap water having δ^18^O values between −10 and −5 ‰. The correction by Equations (1) and (2) using Equation (6) (based on δ^2^H analysis of diluted must) effectively corrects the data for all four dilutions. In any case, a correction is needed, especially for solutions having δ^18^O ranging from −7 to −3 ‰, as the (D/H)_I_ and (D/H)_II_ uncorrected values are lower than the original data of the must by up to 2.3 ppm for (D/H)_I_ and up to 6 ppm for (D/H)_II_. 

## 3. Materials and Methods

### 3.1. Samples

The fermented solutions analysed in this study used brown sugar (El Cibao brand, origin Central/South America), chard sugar (Italian origin), grape sugar (MCRS, Crystal dextro grape), and three authentic grape musts from different Italian areas, diluted with tap water.

### 3.2. Preparation of Water Samples for Dilution

Tap water (δ^18^O = −9.9‰; δ^2^H = −60‰) was evaporated (three days at 100 °C) in order to obtain water with δ^18^O = +10.1‰ and δ^2^H = −5‰. The two waters were mixed in different proportions in order to obtain water samples with δ^18^O of −5‰, 0‰, and +5‰ and δ^2^H of −49‰, −32‰, and −25‰. 

The δ^18^O isotopic values of the evaporated water were higher than those of tap water because during the evaporation process, the lighter isotope (^16^O) is removed to a greater extent than the heavier one (^18^O) [16].

### 3.3. Preparation of the Samples to Submit to Fermentation

One 225 g aliquot of each of the 3 sugar samples was diluted with the 5 water samples up to 1 L, in duplicate, obtaining a total number of 30 solutions.

An aliquot of the 3 samples of must was concentrated by rotavapor (Büchi Rotavapor R-215 with Vacuum pump V-700) at 40 °C with a pressure of 30 milliBar up to a concentration of 50°Brix, to obtain samples of concentrated must (CM).

Aliquots of each of the 3 concentrated must (CM) samples were diluted with 4 water samples (with δ^18^O from −10 to +5‰ and δ^2^H from −60‰ to −20‰), to finally obtain 4 samples of each of the 3 different musts, for a total number of 12 samples, with a sugar concentration of about 200 g/L. 

### 3.4. Samples Fermentation and Distillation

Sugar solutions (30 samples), musts (3 samples), and diluted concentrated musts were fermented for 5–6 days in flasks with inoculation of dry yeasts (Saccharomyces cerevisiae, Enoferm A, Polsinelli). Yeast autolysate extract (OXOID LP0021, Thermo Fisher Scientific) was added as a source of nitrogen, as well as water-soluble B vitamins to facilitate the fermentation of the sugar solutions.

At the end of fermentation, each sample was distilled according to the MA-AS311-05 OIV method using a Cadiot column in order to obtain ethanol with an alcoholic degree higher than 92% Vol.

### 3.5. Analysis of the Samples

The δ^18^O and δ^2^H values of water were analysed in sugar solutions and in diluted concentrated musts before fermentation. Furthermore, the (D/H)_I_ and (D/H)_II_ of ethanol were measured in the distilled ethanol of all the samples, including must, by SNIF-NMR.

(D/H)_I_, (D/H)_II_, R were determined by the OIV-MA-AS-311-05 official method using SNIF-NMR (Site-specific Natural Isotope Fractionation-Nuclear Magnetic Resonance) (FT-NMR AVANCE III 400, Bruker BioSpin GmbH, Rheinstetten, Germany). According to the method, the D/H values were expressed in ppm. The analytical uncertainty of (D/H)_I_ and (D/H)_II_ of sugar solution and grape must measurements calculated with a k factor of 2 was < 1.1 and < 3.7 ppm, respectively.

The analysis of ^18^O/^16^O in water (δ^18^O ‰) was performed with the OIV MA-AS2-12 official method using an IRMS (Isotope Ratio Mass Spectrometry) (SIRA II-VG ISOGAS, Middlewich, UK) connected to an Isoprep 1 (VG Fisons) water/CO_2_ equilibration system.

The analysis of ^2^H/^1^H in water (δ^2^H ‰) was performed with the method adapted from Dhur et al. [17], using an Isoprime© Isotope Ratio Mass Spectrometer connected to a Multiflow© system (Elementar, Hanau, Germany). Briefly, 200 μl of water was pipetted into reaction vessels, where Hokko bead platinum catalysts (Isoprime) were placed to catalyse the equilibration of H_2_ with H_2_O. The vessels were then attached to an online automated equilibration system (Rivoira, Milano, Italy), filled with He containing 10% H_2_, and left to equilibrate for 4 h at 40 °C.

The ^18^O/^16^O and ^2^H/^1^H ratios measured using IRMS were expressed as delta per thousand (δ^18^O and δ^2^H ‰), as the deviation of the isotope ratio of the sample from the V-SMOW (Vienna-Standard Mean Ocean Water, IAEA-International Atomic Energy Agency, Vienna, Austria) international reference standard, on a scale normalised by assigning the δ^18^O consensus value of −55.5‰ to V-SLAP (Standard Light Antarctic Precipitation, water, IAEA). Two in-house working standards (tap water) bracketing the values of the study and calibrated against V-SMOW and V-SLAP were periodically used to calibrate the measurements. The analytical uncertainty of δ^2^H and δ^18^O measurements calculated with a k factor = 2 was < 3 and < 0.3 ‰, respectively.

## 4. Conclusions

This study found that the correction equations quoted in the official OIV method for the values of (D/H)_I_ and (D/H)_II_ of the ethanol from concentrated must (Equations (1) and (2)) are needed in order to obtain reliable results. They provide for correct results in spite of the isotopic composition of dilution water only when δ^2^H is measured (calculation of ${\left(\frac{D}{H}\right)}_{W}^{S}\;$ with Equation (6) which is not an official OIV standard). If δ^18^O is measured (calculation of ${\left(\frac{D}{H}\right)}_{W}^{S}\;$ with Equation (5)) and δ^2^H is computed on the basis of the Global Meteoric Water line (Equation (4)), the corrected values are reliable only when using dilution water that has not suffered isotopic fractionation due, for example, to evaporation (in this case with δ^18^O from −10 to −5 ‰). The isotopic fractionation that occurs during evaporation causes a deviation of the correlation that exists between the δ^18^O and δ^2^H of water as compared to GMWL.

In any case, at least in Europe, generally, the diluted must has values lower than −5‰, because, tap water has values lower than −5‰. Thus, the analysis of δ^18^O in the majority of cases should be enough for a reliable correction of the data. When δ^18^O of the diluted must is higher than −5 ‰, δ^2^H should be analysed.

## Figures and Tables

**Table 1:** δ^18^O and δ^2^H values of diluted sugar samples and of deuterium and hydrogen ratios (D/H)_I_ and (D/H)_II_ of ethanol obtained after fermentation of the three different sugars without correction and after correction using δ^18^O and δ^2^H.

Cane Sugar								
Sample	δ^18^O	δ^2^H	(D/H)_I_	(D/H)_I_	(D/H)_I_	(D/H)_II_	(D/H)_II_	(D/H)_II_
ID	Water Dluted Soltion	Water Dluted Soltion	Ethanol Not Corected	Ethanol Corrected with δ^18^O	Ethanol Corrected with δ^2^H	Ethanol Not Corected	Ethanol Corrected with δ^18^O	Ethanol Corrected with δ^2^H
	(‰ vs. V-SMOW)	(‰, vs. V-SMOW)	(ppm)	(ppm)	(ppm)	(ppm)	(ppm)	(ppm)
**1a**	−10.0	−63.4	106.5	108.6	108.4	120.5	129.2	128.2
**1b**	−10.0	−60.6	106.5	108.7	108.3	120.3	129.1	127.7
**2a**	−5.1	−50.1	106.9	107.8	108.4	122.5	126.5	128.6
**2b**	−5.0	−48.6	107.2	108.1	108.6	123.2	127.1	129.1
**3a**	−0.1	−33.6	107.0	106.8	108.0	124.4	123.6	128.5
**3b**	0.0	−29.4	107.4	107.2	108.3	124.6	123.7	128.2
**4a**	5.1	−24.2	107.3	105.9	108.0	126.0	120.0	128.9
**4b**	5.2	−26.7	107.7	106.2	108.5	126.1	120.1	129.3
**5a**	9.6	−4.9	108.2	105.7	108.3	127.2	116.9	127.8
**5b**	9.8	−5.4	108.3	105.7	108.5	127.5	117.0	128.2
**Beet Sugar**								
**6a**	−9.9	−60.6	91.2	93.3	93.0	122.1	130.8	129.5
**6b**	−10.1	−62.6	91.8	94.0	93.7	122.4	131.2	130.0
**7a**	−5.6	−49.0	92.3	93.4	93.8	124.5	129.0	130.5
**7b**	−5.6	−52.5	92.2	93.3	93.8	124.8	129.3	131.2
**8a**	−0.3	−33.4	92.8	92.7	93.8	126.6	125.9	130.6
**8b**	−0.4	−35.8	92.2	92.0	93.4	126.2	125.7	130.6
**9a**	5.1	−20.1	92.8	91.4	93.4	128.1	122.2	130.5
**9b**	5.1	−19.8	92.9	91.4	93.5	128.2	122.3	130.6
**10a**	9.8	−7.5	92.8	90.3	93.0	128.9	118.4	129.8
**10b**	9.9	−6.1	93.8	91.2	94.0	130.6	120.0	131.3
**Grape Sugar**								
**11a**	−9.5	−57.2	101.6	103.6	103.3	123.8	132.1	130.8
**11b**	−9.6	−58.8	101.5	103.5	103.2	123.4	131.7	130.5
**12a**	−5.0	−49.8	101.8	102.7	103.2	125.6	129.5	131.7
**12b**	−4.9	−44.9	101.9	102.8	103.2	125.7	129.5	131.2
**13a**	−0.1	−26.3	102.8	102.6	103.6	128.2	127.4	131.4
**13b**	0.0	−29.7	102.9	102.6	103.7	128.3	127.3	131.9
**14a**	5.1	−19.7	102.4	100.9	103.0	128.9	123.0	131.3
**14b**	5.0	−20.7	102.7	101.3	103.3	128.9	123.1	131.4
**15a**	9.5	−1.5	102.9	100.4	102.9	129.9	119.7	130.1
**15b**	9.7	−1.0	102.7	100.2	102.7	130.3	119.9	130.4

**Table 2:** δ^18^O and δ^2^H isotopic values of diluted sugar samples and of (D/H)_I_ and (D/H)_II_ of ethanol obtained after fermentation of the three different musts without correction and after correction using δ^18^O and δ^2^H.

Must 1								
Sample	δ^18^O	δ^2^H	(D/H)_I_	(D/H)_I_	(D/H)_I_	(D/H)_II_	(D/H)_II_	(D/H)_II_
ID	Water	Water	Ethanol	Ethanol	Ethanol	Ethanol	Ethanol	Ethanol
	Diluted Must	Diluted Must	Not Corected	Corrected with δ^18^O	Corrected with δ^2^H	Not Corected	Corrected with δ^18^O	Corrected with δ^2^H
	(‰ vs. V-SMOW)	(‰, vs. V-SMOW)	(ppm)	(ppm)	(ppm)	(ppm)	(ppm)	(ppm)
**16**	−7.3	−48.0	99.3	100.3	100.7	123.4	129.5	129.2
**17**	−3.0	−36.6	99.5	100.0	100.6	124.8	126.8	129.2
**18**	2.0	−21.4	99.9	99.2	100.5	126.5	123.5	129.1
**19**	5.0	−10.1	100.3	98.7	100.6	128.0	121.7	129.2
**Must 2**								
**20**	−7.5	−49.0	98.8	100.4	100.3	123.1	129.4	129.0
**21**	−3.0	−33.1	99.1	99.7	100.2	125.0	127.3	129.0
**22**	2.0	−19.4	100.0	99.3	100.6	127.2	124.2	129.6
**23**	5.0	−9.4	100.4	98.9	100.6	129.0	122.8	130.1
**Must 3**								
**24**	−7.2	−45.0	100.6	102.1	101.9	123.9	129.9	129.3
**25**	−3.0	−32.3	101.1	101.6	102.0	125.7	127.7	129.6
**26**	2.0	−18.0	101.1	100.3	101.6	126.7	123.3	128.9
**27**	5.0	−5.9	101.7	100.5	101.9	128.3	123.1	129.0
